# A Flower‐Shaped Thermal Energy Harvester Made by Metamaterials

**DOI:** 10.1002/gch2.201700017

**Published:** 2017-07-27

**Authors:** Wenmei Liu, Chuwen Lan, Muwei Ji, Jitan Yao, Jin Wang, Bo Li, Ji Zhou

**Affiliations:** ^1^ Graduate School at Shenzhen Tsinghua University Shenzhen 510085 China; ^2^ State Key Laboratory of New Ceramics and Processing Tsinghua University Beijing 100084 China

**Keywords:** conversion, energy harvesting, metamaterials, thermal concentration, thermoelectric

## Abstract

Harvesting thermal energy from arbitrary directions has become an exciting theoretical possibility. However, an exact 3D thermal energy harvester is still challenging to achieve for the stringent requirement of highly anisotropic and symmetrical structures with homogenous materials, as well as absence of effective characterization. In this Communication, a flower‐shaped thermal harvesting metamaterial is originally promoted. Numerical simulations imply that heat flux can be concentrated into the target core and a temperature gradient turns out to be more than two times larger than the applied one without obvious distortion or perturbation to the temperature profile outside the concentrator. Temperature transitions of the actual device are experimentally measured to validate the novel structure with consistency of the simulated results with original methods. With ultraefficiency independent of geometrical size, the flower‐shaped thermal harvester facilitates multiple scale energy harvesting with splendid efficient and might help to improve thermoelectric devices efficiency in a totally new perspective.

Solar energy, ocean energy, geothermal and even the wasted heat from the industry were considered as green and reproducible energy for industrial or social requirement.^1^ Therefore, how to collect and utilize this thermal energy more effectively was a significant task and challenge.

Application‐oriented metamaterials,^2^, ^3^, ^4^ exhibiting anomalous and intriguing performances in different physical field,^5^, ^6^, ^7^, ^8^, ^9^, ^10^ were promising candidates for promoting waste heat employment. Heat current was theoretically and experimentally manipulated, controlled, and processed as a medium with artificial materials.^11^, ^12^, ^13^ Thermal cloaks were first theoretically proposed for transient protection of the object from overheating.^14^, ^15^ Thermal concentrators, based on heat current manipulation, provided another prominent alternative for utilizing new energy and improving the energy utilization efficiency with a proper spatial arrangement of natural materials.^16^ The reported annular fan‐shaped thermal concentrating metamaterials^17^, ^18^ were capable of guiding heat flux as required, however, only validated under coplanar condition with line aped or point heat source. Although some theoretical work including 3D heat concentrator has been predicted,^19^ it was investigated experimentally with difficulties in processing and testing. Furthermore, since such thermal concentrators were dependent on rubber or epoxy, they were impossible to operate under high temperature and weak guiding significance for multiple scale practical applications.

Based on spatial structure design, artificially arranged thermal conductivities can be achieved to concentrating heat current with homogenous and isotropic engineering materials. Herein, we pioneered a 3D flower‐shaped thermal energy harvester capable of efficiently harvesting heat in arbitrary directions. Temperature profile transitions were precisely measured with thermocouples. Theoretical identifications, numerical simulations, and experiments of 2D and 3D thermal energy harvesters are presented in this Communication.

With thermal energy efficiently more than double harvested: enhance of temperature gradients and heat flux, numerical and experimental results open up intriguing possibilities in a future all‐in one system, with a wide variety of potential applications to solar thermal panels, thermoelectric generator, and battery or super capacitor type systems.^20^, ^21^

As shown in **Figure** **1**, copper was employed to fabricate the flower‐shaped functional structure, and background medium was made of stainless steel. **Figure** **2**a shows responding simulated results of the temperature profile and heat flux in 2D case. Transformative thermal streamlines indicate that energy density and temperature gradient in inner core were prominently intensified compared to the absence of concentrator. Figure 2b implies that numerical simulation was in agreement with experimental results. More specifically, the magnitudes of these temperature observables were collected by thermocouples (Figure 2c). Distribution of temperature along the observation lines intuitively showed conspicuous transition of temperature fields within thermal concentrator, while temperature fields beyond the concentration cell maintained as original. Temperature gradient was remarkably intensified in concentration core indicating its excellent heat concentrating performance, namely, energy was harvested in the target core by respectable efficient. Therefore, a homogenous and tunable isotropic flower‐shaped thermal concentrator was simply achieved with engineering materials.

**Figure 1:**
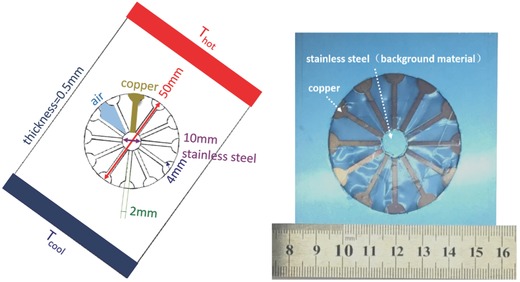
The photograph and geometrical parameters of a fabricated 2D flower‐shaped thermal energy harvester.

**Figure 2:**
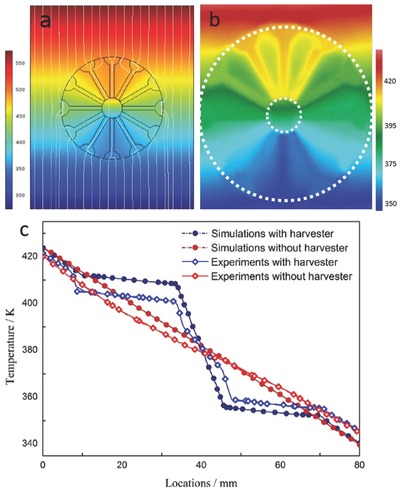
Temperature profile, heat flux, and temperature transitions for 2D flower‐shaped concentrator. a) Simulated results of temperature profile with heat flux represented by white lines. b) Experimental results of temperature profile by infrared imaging devices. c) Simulated and experimental temperature transitions of the energy harvester.

As shown in **Figure** **3**, the considered space, including the inner core (0 ≤ *r* ≤ *a*) and exterior region (*r* ≥ *b*), was made of a homogenous and isotropic background material with thermal conductivity of κ_1_, while a homogenous host material with its thermal conductivity of κ_2_ was employed to the flower‐shaped functional structure region (*a* ≤ *r* ≤ *b*). Petal elements of the flower‐shaped functional structure were uniformly distributed with flexible quantities (*m*). Stable plane heat source (*T*_1_) and cool source (*T*_2_) were employed to the boundaries of an arbitrary direction to acquire a temperature difference Δ*T* = *T*_1_ − *T*_2_ and an applied temperature gradient Grad *T*_0_. To achieve the high degree of anisotropy required for superior flux concentration.^1^

**Figure 3:**
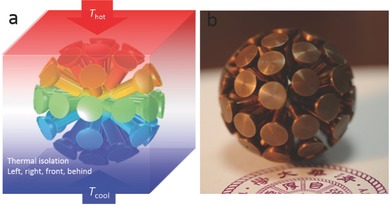
a) The schematic shows spatial structure of a 3D flower‐shaped thermal energy harvester and experimental method for clarity. b) An actual devices for experimental realization.

For 0 ≤ *r* ≤ *a* (inner core) and exterior region (*r* ≥ *b*)

(1) $$\kappa_{r}=\kappa_{\theta }=\kappa_{\varphi }=\kappa_{1}$$

For functional region (*a* ≤ *r* ≤ *b*)

(2) $$\kappa_{r}\to \infty ,\;\kappa_{t}=\kappa_{\theta }=\kappa_{\varphi }\to \;\;0$$

If *κ_2_* > κ_1_ > κ_air_ and *m* is large enough simultaneously, Equation (2) can be exactly satisfied. Here, frequently used engineering materials stainless steel (κ_1_ = 16) and copper (κ_2_ = 377) were employed to background material and host material, respectively, with *m* = 54, *a* = 10 mm, and *b* = 25 mm. COMSOL Multiphysics was applied to invest temperature gradient and thermal flux transitions with Equations (3) and (4)

(3) $$\rho C_{\rho }u\cdot \nabla T\;+\;\nabla \cdot \;q\;\;=\;\;Q\;+\;Q_{\mathrm{ted}}$$

(4) q  =  −κ ∇T

**Figure** **4**a shows the simulated temperature profile and the heat flux streamlines of the thermal energy harvester Ambient thermal flux was obviously compressed into the target inner core and the energy density in the inner core was considerably enhanced by precisely manipulating the local flow of the given thermal concentrator. Meanwhile, thermal field of exterior region kept ideally undisturbed. We also found that the temperature gradient of inner core was prominently increased by 150% compared to the applied one. In case parameters *a*, *b*, and *m* were an ideal match, a perfect thermal concentrator with excellent geometric flexibility to scale up and down and desirable efficiency could be realized.

**Figure 4:**
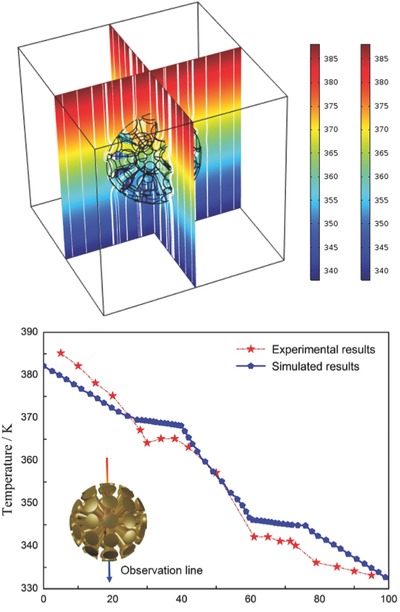
a) Simulated temperature profile and heat flux streamlines of 3D flower‐shaped thermal energy harvester. b) Simulated and experimental temperature transitions of the energy harvester.

Thereafter, experiments were conducted to verify the simulated results. Background material, host material, *a*, *b*, and *m* were, respectively, in line with numerical simulations. Plane heat source and cool source were set as *T*_1_ = 393.15 *K* and *T*_2_ = 323.15 *K*, respectively, with thermocouples lined up along the observation line to verify the validity of temperature gradient transformation in functional regions.

Figure 4b shows the temperature distribution of the actual device. The temperature gradient increased by 130% in target inner core in close proximity to simulated results, while temperature profile in the exterior region was almost constant.

Furthermore, being different from reported work,^17^, ^19^ a fair amount of flexibility of selecting proper materials including metals, polymers, and other materials was promising for applications in a variety of temperature and environment conditions. Although the optimization of temperature gradient and energy density amplification was beyond the scope of this experiment, desirable efficiency of the flower‐shaped thermal concentrator could be accomplished with optimum materials and appropriate geometric size in certain cases.

The 3D flower‐shaped thermal energy harvester is of perfect symmetry, thus apt to harvest thermal energy from arbitrary directions. With thermocouples to measure temperature profile precisely, experimental and numerical results mutually agree with each other well. The temperature gradients transformation implies that it opens new vistas in improving efficiency of thermoelectric devices in circumstance of constrained *ZT* values, the ability of a given material to efficiently produce thermoelectric power, which depends on the Seebeck coefficient S, thermal conductivity κ, electrical conductivity σ, and temperature T. Enhancing temperature difference and heat flux density in unchanged distance. With combination of a 3D flower‐shaped thermal energy harvester and thermoelectric devices, waste heat can be harvested, converted, and stored efficiently.

This type of thermal guiding and energy concentrator may also find applications in devices such as solar cells and biotherapy where high energy density or greater temperature gradient with uniform distribution plays a critical role. It also opens up intriguing possibilities in a future all‐in one system.

We originally promoted a 3D flower‐shaped energy concentrator with widely used engineering materials, which greatly facilitates the thermoelectric generator and other energy harvester, and validated it with numerical simulations as well as experiments. Engineering materials with different constant heat conduction were employed to the flower‐shaped spatial structure, therefore they exhibited overall heat conduction anisotropy and consequently harvested energy in a target core. Simulations and experiments consistently demonstrated its excellent concentration efficiency with remarkable energy density increase and the temperature gradient intensifies in the target core, while temperature profile keeps undisturbed. It should be noted that with judiciously selected materials, efficiency and geometrical size could be tailored to adapt to different conditions. Educed to 2D case, that spatial structure‐oriented thermal concentrator was exactly active. With its extraordinary structure‐oriented and heat conduct‐oriented properties, flower‐shaped thermal concentrator may find potential applications in devices such as solar thermal panels, thermoelectric generator, and miniature heat therapy instruments. A combination of the 3D flower‐shaped thermal energy harvester with thermoelectric generators may open new vistas in future all‐in one systems.

## Conflict of Interest

The authors declare no conflict of interest.
